# Letter from Vienna

**Published:** 1882-04

**Authors:** Frederick Peterson

**Affiliations:** Vienna, Austria


### Correspondence.

VIENNA, AUSTRIA, March, 1882.

*Editors Journal:*—After six months' residence in Vienna and a winter of work, I feel able and at leisure to write another letter for the JOURNAL, and this time about the University of the Austrian capital. Founded in 1365, and with the exception of those of Prague and Heidelberg, the oldest of German universities, it is now attended by over four thousand students, one-third of whom are medical. It was not until the reign of Maria Theresa, however, that Vienna began to be, through the repute of the physician Van Swieten, one of the most popular medical educational centres. But it was during the last two or three decades, that it achieved its greatest reputation through such men as Rokitansky, Hebra and Skoda, and their deaths mark the beginning of its decline; for since that time an economic and short-sighted ministerium has failed to call famous men to fill vacant places, as it well might do, but in some instances has chosen men of as yet very little note. A good example is the recent call of Kundrat of Gratz to fill the chair of Pathology left vacant by the death last year of Heschl, the successor of Rokitansky, when there are so many pathologists, such as Klebs of Prague, Rindfleisch of Würzburg, Von Recklinghausen of Strassburg, Arnold of Heidelberg, and Cohnheim of Leipzig, any one of whom would by acceptance of the position do honor to the University. As it is now, the University honors whom she chooses, for she has the reputation which a number of her professors have not. Among the one hundred and twenty professors and private docents at present in active duty are few of very great fame. In the different departments are the following of more or less celebrity: Since the retirement of Hyrtl, who lives in a villa near the city, no one of importance occupies the chair of Anatomy. Von Brücke in the department of Physiology is second to no one but the Russian Purkinje. In Pathology are Weichselbaum and Hans Chiari, private docents, one of whom will be chosen to go to Gratz, and from April on, Kundrat. In Experimental Pathology is the well-known Stricker. In Internal Medicine are Bamberger and Adalbert Duchek, who died a few days ago at the age of fifty-eight. Surgery is represented by Billroth and Albert. On the Ear are Pollitzer and Urbantschitsch; on the Eye, Arlt and Stellwag. In Gynecology and Obstetrics we have Carl and Gustav Braun, Spaeth, Rokitansky, Jr., and Bandl. On Children's Diseases are Widerhofer and Monti. Kaposi, the son-in-law and also successor of Hebra, has charge of the clinic for Skin Diseases, and Hebra, Jr., and Neumann for Syphilis. The lectures in Psychiatry are given by Theodore Meynert and Leidesdorf; in Medical Chemistry, Ludwig; Laryngoscopy, Schrötter and Störk; Urinary Organs, Ultzmann.

Of all these perhaps the best known in America, and indeed throughout the world, is Theodore Billroth, who, first known as an excellent Pathologist, has of late claimed the attention of medical men through his surgical achievements. A few months of attendance at his neat and well-appointed clinic, with seating capacity for two hundred or more students, is certainly no loss of time, nor is a walk on Tuesday mornings through his wards with him. At the beginning of the session one of the first things shown us was the successful case of excision of pylorus for carcinoma—a woman about fifty years in very good health and with no sign of return of cancer. The operation had been performed some six months before. He then showed us the stomachs of the others on whom he had performed the operation with fatal result, and explained his method. Gastrotomy for œsophageal stenosis is quite often performed. The first step is to open the abdomen and secure the adhesion of stomach wall to the opening, and in a few days the operation is completed by the opening of that organ. That is a way, too, they have of making a suprapubic lithotomy in case of cystitis, to prevent infiltration of purulent urine and septicemia—to open as far as the bladder and wait until the fistula sides are healed before proceeding farther. Carcinoma is unusually common in Vienna—of breast, uterus, stomach, tongue and œsophagus. The removal of a tongue is almost an every-day occurrence at Billroth's clinic. He generally cuts down upon and ties the lateral lingual artery on each side before the removal of the organ. One is surprised to see so much goitre here, and Billroth removes thyroid glands very often, and with remarkably good results. For example, a boy of twenty and a woman of fifty, on whom the operation required seven minutes each, I saw walk into the clinic, the boy in seven days, the woman in six days after it, the wounds entirely healed by first intention. The incisions are made obliquely from the neighborhood of the sterno-cleido-mastoids to meet in the trachea, thus avoiding the deformity which a large scar in the median line is apt to cause by contracting. He showed us also ten cases from the city on whom he had operated within a short time, all perfectly well. His laparotomies are performed on Saturdays, and are only to be seen by those receiving printed invitations, which he sends preferably to foreigners. The conditions of your admission are that you have not been near the pathological institute that day, or near any zymotic disease, and that you appear in a complete change of apparel from what you have worn during the week. The anæsthetic in use here, as indeed in all the clinics in the hospital, is as follows:

℞ Chloroformi, 500 grammes.
Etheris sulph.
Alcoholis, ā ā 150 grammes.

This is poured upon a small piece of flannel stretched over a wire frame large enough to cover the nose and mouth of the patient. They claim to reduce the danger of death from narcosis to a minimum by use of this mixture. The carbolic acid spray is rarely or never used, but wounds are washed and hands of operators and instruments are bathed in 1 per cent. or 3 per cent. solution of carbolic acid. All wounds, no matter where they are, and in all the hospital clinics and wards, are treated with iodoform, and with splendid results. The iodoform powder is rubbed into gauze as we rub plaster of Paris into our bandages. This gauze is applied directly to a wound after freely dusting the same with the powder; over the gauze comes a large pad of absorbent cotton, and over this a large piece of oiled silk or jaconnet; then the bandage. Iodoform is a good deodorizer, and I have seen the gauze strips taken from the wound of an œsophagotomy after an eight days' stay with not the slightest odor, and the same from a collum uteri after removal of a carcinoma. Prof. Luecke of Strassburg is introducing naphthol in its place as a much cheaper and equally effective application. Withal, Prof. Billroth, in spite of his great knowledge and attainments, has not the faculty of imparting it well to others. He is a type of the sturdy, stout German with whom life has gone well, is about sixty years of age, and wears a gray, full German beard. He speaks so low generally that the most of the sound is lost in his mustache and whiskers. Prof. Albert, who is much younger, has all the ability of Billroth as an operator, and is a teacher who arouses enthusiasm in all who hear him. It is more instructive to attend his clinical lectures on surgery than those of Billroth. Among the operations they have performed during the winter semester, beside such as above mentioned, are tracheotomies, lithotomies, litholapaxies, amputations, resection of joints, rhinoplasties, staphyloraphies, ligations of subclavian and lesser arteries, removal of tumors from antrum highmorianum, etc. One case of Billroth's may prove interesting—which was to have been an ovariotomy on a woman of fifty, but which revealed after the exploratory incision a large medullary carcinoma of abdominal glands and omentum majus. Parts of this were removed and the abdomen sewed up. That was several months ago; and the woman walks about the wards in good health, of course eventually to succumb to her fatal disease.

For the obstetric student Vienna presents a good field. This department of the Allgemeines Krankenhaus occupies the seventh, eighth and ninth courts, and is divided into the three clinics of Spaeth, Carl Braun and Gustav Braun. It is noteworthy to state that there are three brothers Braun and one son-in-law, all well known as gynecologists. In the three clinics together the average number of births daily is between thirty and forty—enough for an athletic student. Almost all of the obstetric operations are performed in the course of a semester. Natural labors are not brought before the class, except at Spaeth's, which is more for beginners. At Braun's clinic during the winter have been a large number of forceps deliveries, turnings, several cephalotomies, operations for extra-uterine pregnancy, several ovariotomies, one successful Cæsarian section, four or five complete removals of uterus per vaginam for carcinoma, and any number of operations for ruptured perineum, and fistulas, vesico-vaginal, recto-vaginal, ureto-vaginal, urethra-vaginal, and vagino-peritoneal. A curious cause for a large fistula between vagina and pouch of Douglas in one case was coitus. Braun rightly calls the vagina the Pandora box of woman's ills. The Swedish massage system is in great vogue for the treatment of fistulas before operation, for perimetritis and oophoritis. Iodoform also comes into use here. All wounds require that dressing. After an operation for ruptured perineum, in addition to the plenteous sprinkling by the teaspoonful upon the seam, iodoform in stick form is laid in the vagina and rectum; then over the whole absorbent cotton, jaconnet and bandage. No ill effects follow the use of iodoform, except if extremely long continued, when headaches are complained of, and occasionally more serious symptoms of iodine intoxication supervene.

The clinical lectures of Meynert on Psychiatry are full of interest. He has one or two wards in the General Hospital, to which for his daily teaching patients from the large Insane Asylum, five minutes' walk away, are brought. The various forms of nervous diseases are exhibited to the class and thoroughly examined and explained.

Perhaps no other city in the world affords the material for the study of Pathology which Vienna does. Every morning from six to ten post-mortem examinations are made in the Pathological Institute connected with the Hospital; while the immense Poorhouse across the road, the Garrison Hospital for the use of the military students close by, the large St. Anna's Children's Hospital, the Foundling Asylum, and Insane Asylum, all close together, provide an enormous quantity of material for pathological and anatomical purposes. It is no wonder that the museums in the various departments are so rich. In making these post-mortem examinations there is no "unanimous agreement not to open the head," but everything receives thorough research from keen, scientific and unfailing eyes.

To non-professional people the Ring Theatre fire is already a thing of the past; but to doctors it still teaches something, and I may therefore be pardoned for referring to it. A committee of physicians, Eduard Hofmann, the eminent professor of Medical Jurisprudence, at its head, was appointed to identify bodies. Nearly four hundred bodies were brought to the city morgue, which is the Pathological Institute, but never before had its accommodations been so taxed. It has wards for perhaps a hundred dead; so those who came in that one fatal night in December were laid along the halls a few feet apart on both floors. This committee by arduous labor was able to identify two hundred and fifty. Legal obductions were made upon three only. Spectrum analysis of the blood gave the characteristic spectrum for poisoning by carbonic oxide, which, added to the fact that few bodies presented the erythema and vesicles of burns intra vitam, showed conclusively that suffocation by carbonic acid and smoke and poisoning by carbonic oxide were the causes of death, while the frightful burning of the bodies occurred post-mortem. It was rare that sex could be determined by external organs, and the uterus and ovaries had to be sought for for this purpose. Age was determined to some extent by examination of epiphysis of humerus, separate until twenty-fourth year, by condition of ovaries, and by progress of calcification in larynx and ribs, beginning in thirtieth and ending in fortieth year. The general appearance of the bodies was hideous: the outstretched arms as if to ward off something, the dreadful trismus which even involved the palpebral muscles, the red foam at the lips, the completely blackened features, and the general disfigurement of the mangled and shapeless bodies. Some idea of the heat may be obtained when I say that it was so great as to burst open the skull and cook the brains; that in almost all cases the abdomen was burst open by its action upon the evaporative fluids of the body; that sometimes a corpse was so shrunken that little seemed left except the spinal column; that the albuminous fluids of the eye were completely coagulated; and that bones and teeth were often so calcined as to crumble at the touch. The delicacy of a hand would sometimes reveal the sex. In several cases hernias and trusses, in one case coxitis, led to identification. Six women were pregnant, two in sixth, two in third, two in second month, and of the first two little was left of the women except the round black ball of the uterus and charred vetebræ and pelvis. The bodies were buried as soon as possible, as the air was so polluted by the odor as to render it dangerous for the hospital. From the three hundred bodies whose ashes still lie in the ruined auditorium of the theatre arose such a pestiferous vapor that the city ordered it immediately disinfected with carbolate and phenylate of lime.

The season has passed over so sunny and smiling that the Viennese call it an Italian winter. The result has been an increase in all zymotic affections; small-pox is an all-the-year-round disease. Indeed, last year an American student caught it at the Poliklinik and died here. The report last week gave one hundred and fifteen cases in the city small-pox hospital and as many more in the suburban towns, together with plenty of typhus exanthematicus and diphtheria, while scarlatina, typhoid fever and measles are epidemic. It has been conclusively demonstrated by the Vienna physicians that those who drink the water supplied by the aqueduct from the Schneeberg, thirty miles away, do not have typhoid fever, while those who drink well water are the ones who receive the contagion. As the well-water districts are Dornbach, and other of its suburbs, entirely separated from those supplied from the Schneeberg, there is no room for mistake.

Before closing, something may be said for the benefit of students who are coming this way. Except London, Vienna is the most expensive city to live in in Europe. The bright side for the student is that there is such a vast supply of material compressed into such small space, for the Krankenhaus with its three thousand patients, the Children's Hospital, the Garrison Hospital, the Insane Asylum, the Poorhouse, the anatomical, chemical and pathological laboratories, and the Poliklinik all stand close together. The dark side of this is that, owing to the opulent and luxurious decline of the University at present, all this material is put into the hands of the assistant physicians, or internes, a brood of vampires who prey upon the foreign students. From the professors themselves little is to be obtained, except excellent lectures over the patients. For actual examination of patients yourself you must seek the assistants. Their courses cost from $5 to $25 per month. The courses on the Eye and Ear are comparatively inexpensive. Under Profs. Monti, at the Poliklinik, and Widerhofer, at the Kinderspital, one is sated with children's diseases at trifling expense. But courses in laryngoscopy, and other departments of physical diagnosis, in obstetric operations and gynecology, in surgery, cost from $10 to $25 per month. One who does not undertake too much will not need to pay out for instruction more than $30 per month. With economy one can live in Vienna for $30 per month. The best plan for a student is to take a room as much beyond a ten-minute walk from the Krankenhaus as he has time to spare (for all the Hausfrauen within that distance look upon Americans as bonanzas), to have his breakfast and supper in his room, and take his dinner at a restaurant. He will look quite in vain for a pension. Vienna is much more expensive than Berlin, and about double as expensive as other German universities of great reputation, such as Leipzig, Strassburg, Heidelberg, Prague and Würzburg. During the semester some one hundred and fifty Americans have been attending lectures here, beside a large number of Scotchmen and a few Englishmen. Indeed, it has gone so far that Hebra, Jr., lectures in English. Most of the foreigners seem discontented here by their somewhat Jewish reception, and it is probable that the number of Americans, as well as of students of other nations, will gradually decrease each year, unless the Ministerium takes greater pains to uphold the reputation of the University. One thing is true, the University can at any time regain its former supremacy.

It is a false idea some have of railing at German theories. I have some respect for a medical theory founded on twenty years' experience and study of thousands of cases. Theory, of course, is not truth, but is founded on truth, and is a precursor of other truths. Really the Germans are a most practical people, as practical as theoretical. They do not, at least, found an absurd theory on ten hours' observation, but their theories are the results of life-times of observation. The fanatical London professors tell the students coming here, by all means avoid the Germans, do not listen to them, only look at their cases; and I know a young Englishman who is in a state of continual trepidation lest he should catch a German idea. Myself I have no doubt that he has been so thoroughly inoculated with weak English theories that his mental constitution is proof against the German theory epidemic.

I was reading a few days ago of the Veterinary Department of the University and Horse Hospital. To obtain a diploma from this school one must attend six semesters, averaging five months each, or twice, nay thrice even the amount of study required of American physicians. Let no one say invidiously that the Germans value their horses more than we do human beings! FREDERICK PETERSON.

